# A lightweight DSD-UNet-based method for clustered litchi segmentation

**DOI:** 10.3389/fpls.2026.1855288

**Published:** 2026-08-11

**Authors:** Zhiping Tan, Yihao Huang, Dapeng Ye

**Affiliations:** 1 College of Electronics and Information, Guangdong Polytechnic Normal University, Guangzhou, China; 2 College of Mechanical and Electrical Engineering, Fujian Agriculture and Forestry University, Fuzhou, China

**Keywords:** clustered litchi, deep learning, dual cross-attention mechanism, image segmentation, lightweight model

## Abstract

Automated litchi harvesting demands clustered fruit segmentation with both high accuracy and real-time performance, yet high computational complexity and parameter redundancy pose critical bottlenecks for practical deployment. To tackle these challenges, we propose a lightweight DSD-UNet method for fast and high-precision segmentation of litchi clusters. The DSD-UNet incorporates depthwise separable convolutions to replace traditional convolutional structures, significantly reducing parameters and computational complexity. To further enhance the model’s performance, we introduce a dual cross-attention mechanism into the skip connections,which integrates spatial and channel attention to adaptively strengthen the representation of target regions in feature maps and improve sensitivity to slender stem features. Additionally, we develop a composite loss function (BL-Loss), combining binary cross-entropy with level-set loss, and optimize it using a staged training strategy to alleviate within-foreground pixel imbalance and preserve the boundary continuity of slender fruit–stem connections. Experimental results under varying illumination conditions demonstrate that DSD-UNet achieves a mean Intersection over Union (*mIoU*) of 91.40% and an *F1-score* of 95.42% for the segmentation of clustered litchi, with an inference speed of 15.41 frames per second and a parameter count of only 5.16M. Compared with the original U-Net, our DSD-UNet model improves *mIoU* and *F1-score* by 3.08 and 1.81 percentage points, respectively, increases inference speed by 138.9%, and achieves a 61.5% reduction in parameter count, confirming that the proposed method effectively enhances segmentation accuracy while maintaining model lightweightness. These results underscore the potential of our method to significantly improve automated harvesting processes in litchi production.

## 1 Introduction

Automated litchi harvesting requires precise pixel-level segmentation of fruit-stem structures for cutting path generation. Unlike single-fruit crops, litchi’s clustered growth pattern with slender stems (2–4 mm diameter) presents unique challenges: (1) severe adhesion between fruits and stems under variable illumination and occlusion, frequently leading to fragmented or under-segmented boundaries, particularly under complex orchard canopy conditions; (2) high morphological similarity between fruit-bearing and non-fruit-bearing stems, complicating the identification of true picking targets; (3) computational constraints of mobile platforms requiring lightweight solutions.

Early studies primarily relied on handcrafted features for fruit identification and segmentation (Liu T. et al., 2023). For example, Mao et al. segmented mature litchi image by rotating and selecting the H component in HSV color space, combining fuzzy clustering and Markov random field constraints, achieving an average accuracy of approximately 90% (Mao et al., 2011). Zhuang et al. reconstructed the hue component in the HSV color space and utilized Otsu thresholding for segmenting litchi stems, filtering out non-target stems based on the spatial relationship that the stem centroid is above the connected fruit centroid (Zhuang et al., 2019b). Dorj et al. proposed an image processing-based method for citrus fruit detection and counting, combining RGB-HSV conversion, noise removal, and watershed segmentation to estimate orchard yield (Dorj et al., 2017). However, traditional approaches are highly sensitive to illumination conditions, background complexity, and camera perspective (Ghazal et al., 2024), and exhibit limited robustness to noise and occlusion. Moreover, as they lack the capacity to learn high-level feature representations from large-scale data, they cannot satisfy the demand for fine-grained segmentation in the litchi fruit–stem coupling scenario (Coll-Ribes et al., 2023).

Deep learning, empowered by large-scale data and powerful computational resources, has introduced innovative solutions and new development opportunities for agriculture, delivering remarkable advances in target recognition performance under complex agricultural conditions (Tan et al., 2025). Li et al. enhanced litchi small-object detection using YOLOv7 integrated with the lightweight ELAN-L module, CBAM attention mechanism, and the CoT transformer, achieving high-precision results (*mAP@*0.5 of 98.60%) (Li et al., 2024). In crops such as tomato and grape, studies based on keypoint detection, RGB-D fusion, and multi-stage strategies have further validated the applicability of detection paradigms in field environments (Du et al., 2023). While detection-based methods (Du et al., 2023; Li et al., 2024), achieve high *mAP*, they produce bounding boxes insufficient for precise cutting path definition.

In the field of semantic segmentation, significant advances have been achieved in litchi target segmentation. For instance, Peng et al. proposed ResDense-focal-DeepLabV3+, which combines ResNet and DenseNet backbones with a focal loss function and attained a *mIoU* of 79.7% under challenging canopy conditions (Peng et al., 2023). Wang et al. (Wang C. et al., 2024) and Zhong et al. (2021), using improved YOLACT frameworks incorporating branch morphology reconstruction and the MFBB training strategy, reported localization accuracies of 91.5% and 89.7% for picking points, respectively. These efforts demonstrate that segmentation strategies emphasizing structural relationships and boundary quality can effectively enhance operational reliability in orchard scenarios. Research across different crop species has also provided valuable insights for addressing common challenges, such as slender structures and adhesive boundaries. Liang et al. achieved a localization accuracy of 92.0% for tomato lateral branch pruning points (Liang et al., 2025); Xiong et al. realized semantic mapping in citrus orchards (*mIoU* 79.31%) via VINS-RGBD and BiSeNetV1 fusion (Xiong et al., 2023); Xiang et al.’s MixSegNext attained a *mIoU* of 92.85% for Sichuan pepper targets (Xiang et al., 2025); and Lei et al. developed a method yielding a *mIoU* of 83.5% for strawberry segmentation in complex scenarios (Lei et al., 2022).

Among various segmentation architectures, encoder-decoder structures are especially favored for their ability to effectively fuse multi-scale contextual information. The fully convolutional network (Wang Y. et al., 2024) pioneered end-to-end semantic segmentation; U-Net (Ronneberger et al., 2015), with its distinctive symmetric encoder-decoder architecture and skip connections, demonstrated outstanding segmentation accuracy for fine structures in biomedical image analysis—a capability that has been equally validated in subsequent agricultural vision tasks. For example, Li et al. adopted an improved U-Net model to achieve high-precision segmentation of kiwifruit and fruit stems in orchard environments (Li et al., 2025), while Wang et al. leveraged U-Net for the precise delineation of maize crop rows in field conditions, achieving a *mIoU* of 83.23% (Diao et al., 2023). DeepLabV3+ (Wang and Liu, 2021), by integrating an atrous spatial pyramid pooling (ASPP) module and an encoder-decoder paradigm, has proved highly effective in aggregating multi-scale contextual cues, making it a robust baseline model for complex scenarios. For instance, Song et al. utilized DeepLabV3+ to successfully segment fruit sepals and branches in orchards (Song et al., 2021).

However, a deeper analysis reveals that while current methods prioritize segmentation accuracy, they often overlook the trade-off between model complexity and deployment feasibility. In contemporary agricultural harvesting vision tasks, mainstream approaches frequently rely on two-stage processing pipelines: initially detecting or segmenting the fruit region (Tang et al., 2025), followed by a secondary stage in which the corresponding fruit stem is further matched or segmented.

This class of methods is marked by notable limitations: On the one hand, the two-stage design significantly increases model parameters and computational complexity, hindering their suitability for resource-constrained mobile harvesting platforms, which demand low-latency and low-power consumption, and ultimately raising the cost of edge deployment (Zhuang et al., 2019a). On the other hand, due to their dependence on intermediate result transfer and matching between stages, such models struggle to perform end-to-end learning and exploitation of the holistic shape, texture, and topological features inherent in the fruit–stem structure (Guo et al., 2019), leading to frequent mismatches and segmentation errors.

Comprehensive analysis reveals that existing approaches for fruit-stem coupling segmentation in litchi encounter several core bottlenecks. A primary challenge lies in the ineffective modeling of fruit-stem structures and cross-regional contextual relationships, which leads to poor topological continuity and frequent mis-segmentation when facing complex backgrounds and occlusions. This issue is further compounded by the insufficient robustness of current methods in capturing boundary and texture features of slender, small-scale fruit stems, resulting in a tendency for segmentation discontinuities. In addition, the lack of lightweight designs capable of balancing segmentation accuracy with real-time performance makes it difficult for these approaches to be deployed online on mobile platforms.

To address these challenges and limitations, the ideal solution should offer coordinated improvements in three key dimensions: the adoption of lightweight backbone networks to greatly reduce computational overhead, the integration of effective attention mechanisms to enhance feature discrimination for fine structures, and optimization strategies for boundary continuity to improve segmentation quality at a detailed level. The U-Net architecture—with its parameter efficiency, strong detail preservation, and adaptability for lightweight modifications—emerges as a highly suitable foundation for this goal. The inherent encoder-decoder structure of U-Net ensures parameter efficiency, while its symmetric design facilitates the incorporation of lightweight techniques such as depthwise separable convolutions (Yun et al., 2022). The skip connections preserve shallow feature details, making the U-Net especially appropriate for processing slender fruit-stem structures in litchi. Moreover, its validated capability in biomedical imagery for segmenting fine structures aligns closely with the fine-grained segmentation requirements of litchi fruit-stem coupling.

To this end, we propose a lightweight segmentation model, DSD-UNet, tailored for clustered litchi fruits. Employing an end-to-end single-stage segmentation strategy, the core concept of DSD-UNet is to directly conduct pixel-wise prediction of the fruit–stem ensemble. This unified framework enables both structural perception and segmentation within a single network, thereby avoiding the matching errors and high computational costs associated with traditional two-stage methods and effectively filtering out interference from non-fruit-bearing stems. The main contributions of this study are as follows:

1. Lightweight Architecture: Reconstruction of the encoder using depthwise separable convolutions to replace traditional redundant convolutional structures, reducing parameters by 61.5% while maintaining accuracy.
2. Adaptive DCA Integration in Skip Connections: Building on existing cross-attention mechanisms, we strategically embed a Dual Cross-Attention (DCA) module within the skip connections of U-Net to address the spatial-channel feature correlation of slender fruit stems in clustered litchi. This targeted placement enables effective modeling of long-range dependencies across multi-scale features, thereby enhancing feature discrimination and topological continuity preservation for fine-grained stem segmentation.
3. Boundary-aware Loss (BL-Loss):Hybrid loss combining Binary Cross-Entropy and level-set constraints for topology-preserving segmentation.

The rest of this paper is organized as follows: Section 2 presents the dataset construction process. Section 3 provides a detailed framework description of the DSD-UNet algorithm. Section 4 evaluates the effectiveness of the DSD-UNet algorithm. Finally, conclusions are drawn, and possible future research directions are proposed in Section 5.

## 2 Dataset construction

### 2.1 Data collection

**Figure 1:**
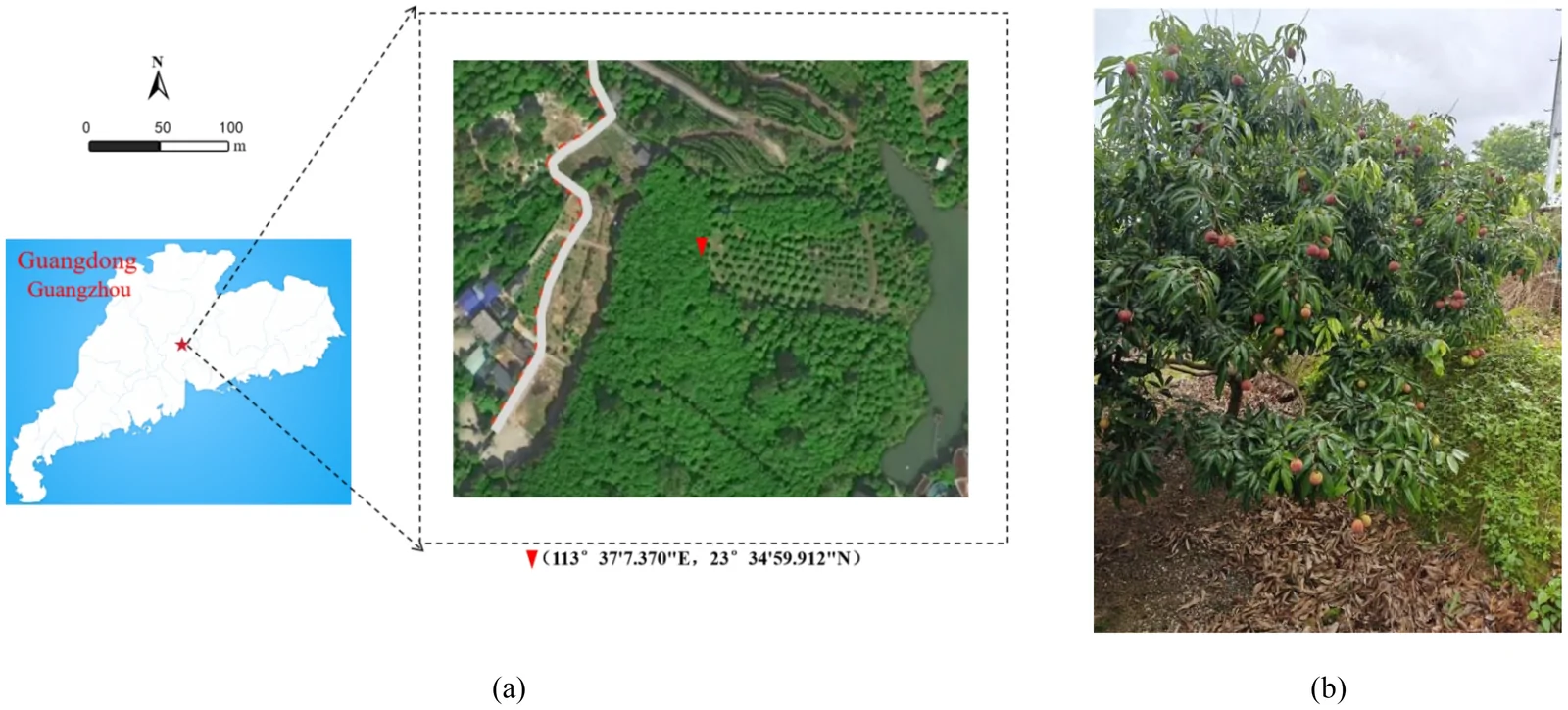
Map of the study area: **(a)** geographical location; **(b)** in-field photos of the study site.

Data were collected from a litchi orchard in Guangzhou during the June 2024 harvest season under varying illumination conditions, as shown in **Figure 1a**. The orchard cultivates the ‘Jinggang Hongnuo’ variety, which is characterized by large, plump fruits and slender, pliable stems. Due to the small ratio of stem diameter to fruit size, the fine geometry and deformability of the stem impose stricter requirements on vision-based precise segmentation, as shown in **Figure 1b**. Data acquisition was conducted on June 24–25, 2024. To obtain image samples under diverse illumination, we collected data on June 24, 9:00–12:00 (rainy, low-light conditions) and 14:00–17:00 (overcast, diffuse illumination), and on June 25, 9:00–12:00 (sunny, strong illumination) and 13:00–16:00 (sunny transitioning to overcast, varying illumination). By covering natural lighting scenarios such as direct strong sunlight, cloud cover, and rainfall, the dataset reflects the complex and variable illumination encountered in real orchard operations, thereby enhancing the model’s adaptability and robustness across lighting conditions. In the orchard, we randomly selected different litchi trees and captured multi-view, multi-scale images using a Canon 600D camera (TAMRON 18–200 mm, f/3.5–6.3) and mobile devices, encompassing frontal views, side views, and zoomed scenes.

The imaging distance between the litchi fruits and the camera was controlled within 0.2–2 m. The acquired images had a resolution of 4096×3072 and were stored in JPEG format. During the litchi harvest in June 2024, a total of 790 valid litchi images were collected. All images depict multi-element scenes comprising fruits, stems, branches, and leaves, and complex orchard backgrounds.

**Figure 2:**
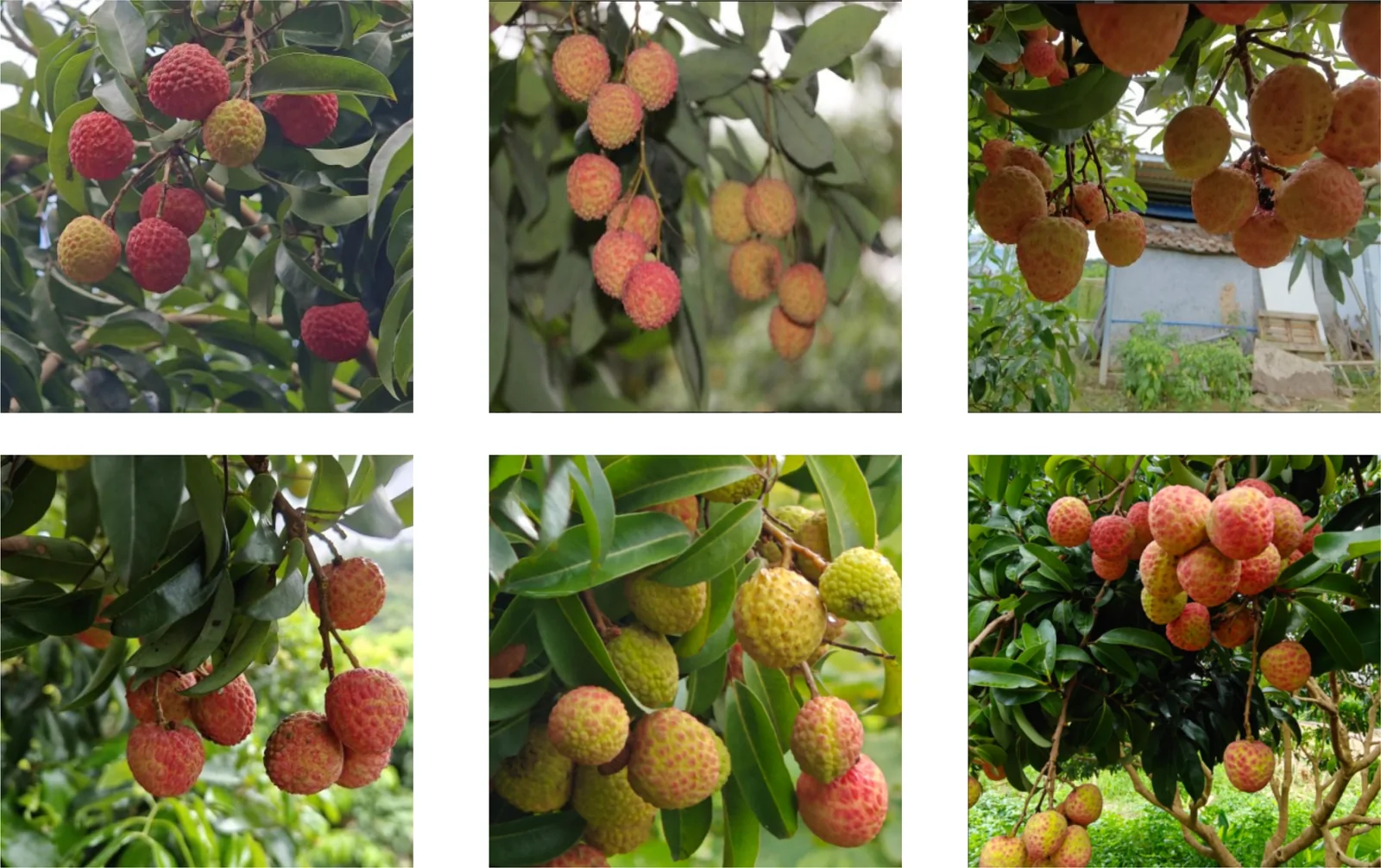
Litchi images collected in the orchard.

As shown in **Figure 2**, litchi fruits grow in clusters within an unstructured orchard environment. Within clusters, fruits exhibit adhesion, overlap, and mutual occlusion, together with additional occlusions from stems and foliage. In addition, the image background contains bare stems without attached fruits and other interfering objects, introducing background interference. The combined effect of the clustered growth pattern and the complex background environment increases the difficulty of accurate segmentation of litchi fruits and stems.

A total of 790 litchi images were collected. Based on illumination conditions, the images were categorized into three classes: non-uniform illumination, low illumination, and sufficient illumination, comprising 186, 126, and 478 images, respectively. The classification was based on image brightness levels, utilizing the brightness histogram of the images: non-uniform illumination was characterized by significant brightness variations, low illumination featured overall dimness often resulting in noise, and sufficient illumination provided a balanced brightness conducive for visual tasks. The dataset was then randomly partitioned at a 7:2:1 ratio into training, validation, and test sets, yielding 540, 150, and 100 images, respectively. Details of the dataset split are summarized in **Table 1**.

**Table 1:** Image count distribution in the litchi dataset.

Datasets	Number of images in different acquisition environments			
	Non-uniform illumination	Low illumination	Sufficient illumination	Total
Training set	127	74	339	540
Validation set	35	31	84	150
Test set	24	21	55	100
Total	186	126	478	790

Although the dataset exhibits non-uniform distribution across illumination conditions, the combined samples of non-uniform and low illumination constitute a substantial proportion comparable to sufficient illumination cases. This distribution naturally reflects the illumination scenarios encountered in actual orchard operations during the harvest season. In real-world harvesting environments, sufficient illumination is more prevalent, while challenging low-light conditions occur less frequently but are critical for model robustness validation. Preserving this natural distribution in the train/validation/test split ensures that the model’s performance metrics reflect realistic operational conditions.

### 2.2 Dataset annotation and augmentation

**Figure 3:**
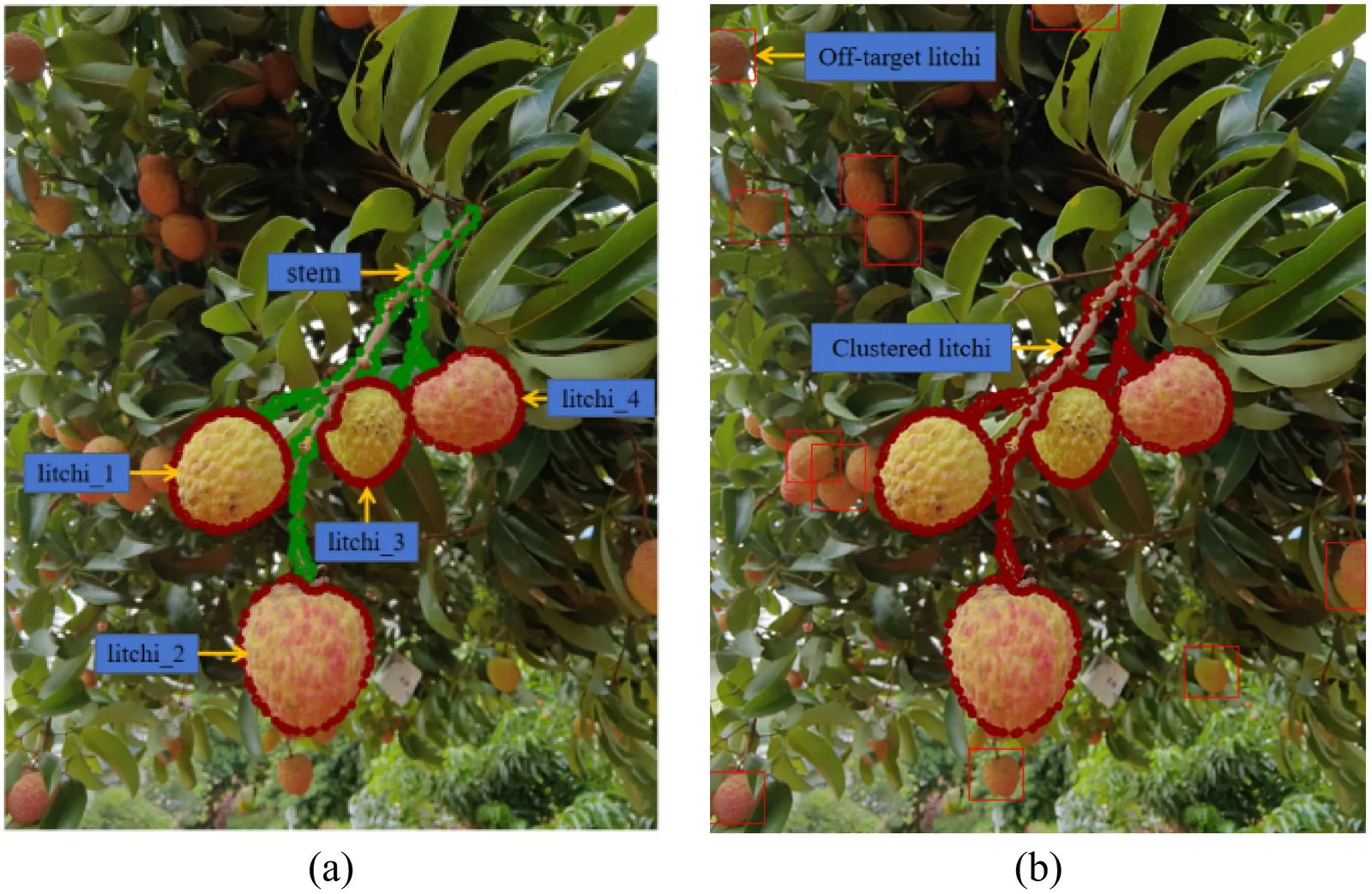
Annotation schemes for litchi fruits and stems: **(a)** conventional separate-class labeling; **(b)** proposed unified fruit–stem class labeling.

The sample images acquired in this study were annotated using Labelme. For each image, segmentation masks were delineated for litchi fruits and their stems, and the corresponding labels were generated. The specific annotation rules are as follows: the dataset was annotated with close-up, fruit-bearing litchi clusters as the target objects. In contrast to the separate labeling of fruits and stems shown in **Figure 3a**, we adopted the unified strategy illustrated in **Figure 3b**, in which each fruit together with its supporting stem is annotated as a single instance of the same class (i.e., a fruit-with-stem instance); within a cluster, every fruit–stem pair is treated as one instance of that class to enhance their shape and texture features and distinguish them from background disturbances. During annotation, only litchi targets with clearly discernible morphology were precisely delineated, whereas fruits and their associated stems that could not be reliably identified due to extreme illumination, severe occlusion, or morphological ambiguity were left unannotated. This strategy ensures that every annotated target within a litchi cluster is a semantic unit that contains the complete fruit–stem connection, yielding an annotated dataset with well-defined boundaries and intact morphological characteristics.

Under this annotation protocol, the segmentation task is formulated as a binary semantic segmentation problem. The foreground class denotes the unified fruit-with-stem region illustrated in **Figure 3b**, and the background class includes leaves, branches, bare stems, and other non-target objects. Fruit and stem are not defined as separate output categories; instead, each annotated instance represents a single connected fruit–stem unit in the ground-truth mask.

To improve the model’s generalization and robustness to visual perturbations, we performed data augmentation via a Python script. Specifically, we applied random horizontal and vertical flips with probabilities of 0.5 and 0.3 respectively, random rotations within the range of ±15, and additive Gaussian noise with mean 0 and standard deviation 0.02 to the 540 annotated training images (DeVries and Taylor, 2017), expanding the training set to 1,080 images in total for model training.

## 3 The proposed algorithm

**Figure 4:**
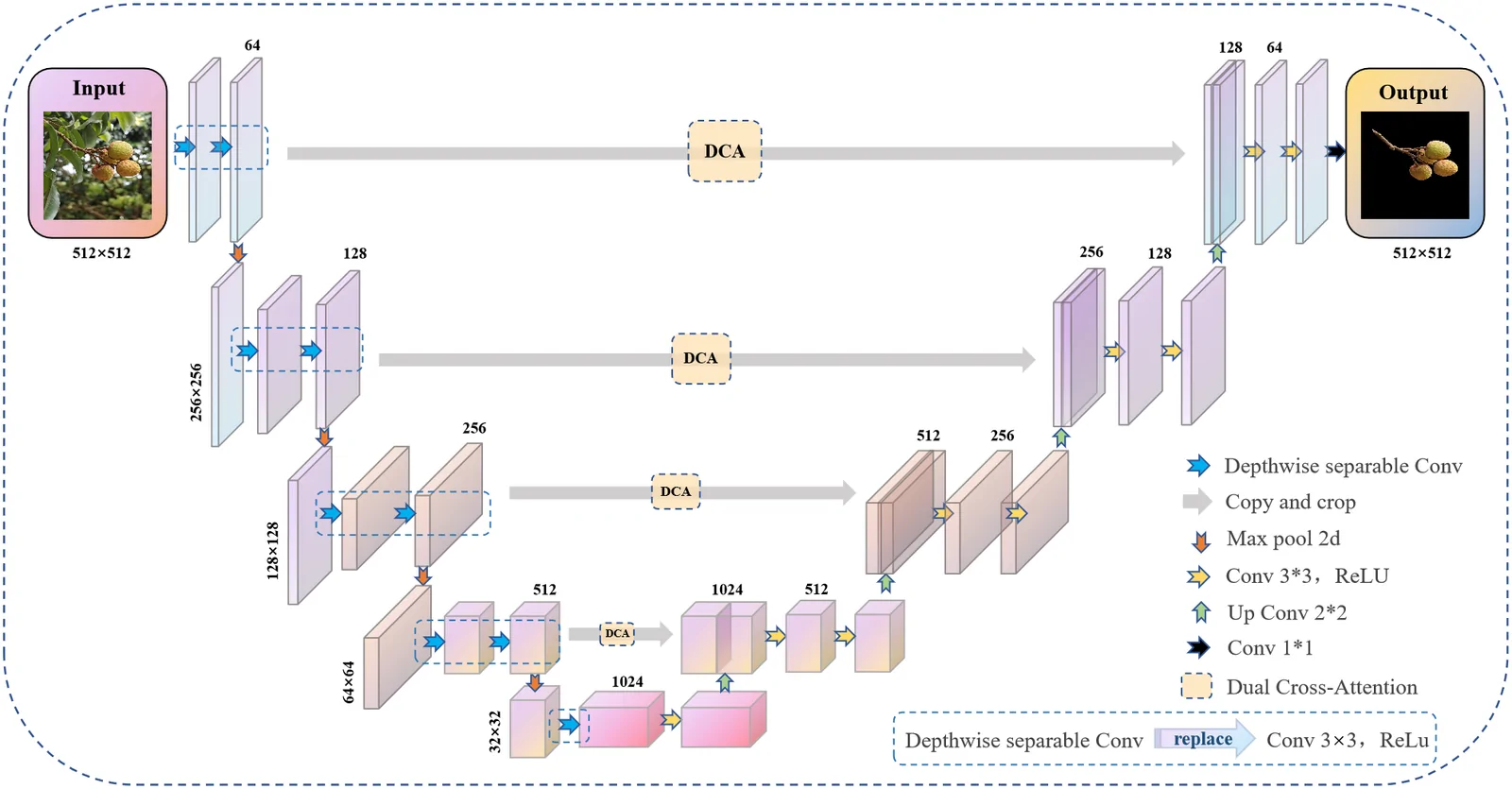
Architecture of the DSD-UNet model.

This chapter addresses the task of integrated fruit–stem segmentation for clustered litchi in unstructured orchard environments by developing a lightweight DSD-UNet model (**Figure 4**). Enhancing the U-Net backbone, our model reconstructs the encoder with depthwise separable convolutions, which reduce computational cost and network redundancy; positions a Dual Cross-Attention (DCA) mechanism strategically within the skip connections to improve spatial-channel feature correlation and boundary delineation for slender stems; and applies a composite BL-Loss function at the prediction stage to address class imbalance and preserve topological continuity. The implementation details are elaborated in Sections 3.1–3.3.

### 3.1 Depthwise separable convolution

**Figure 5:**
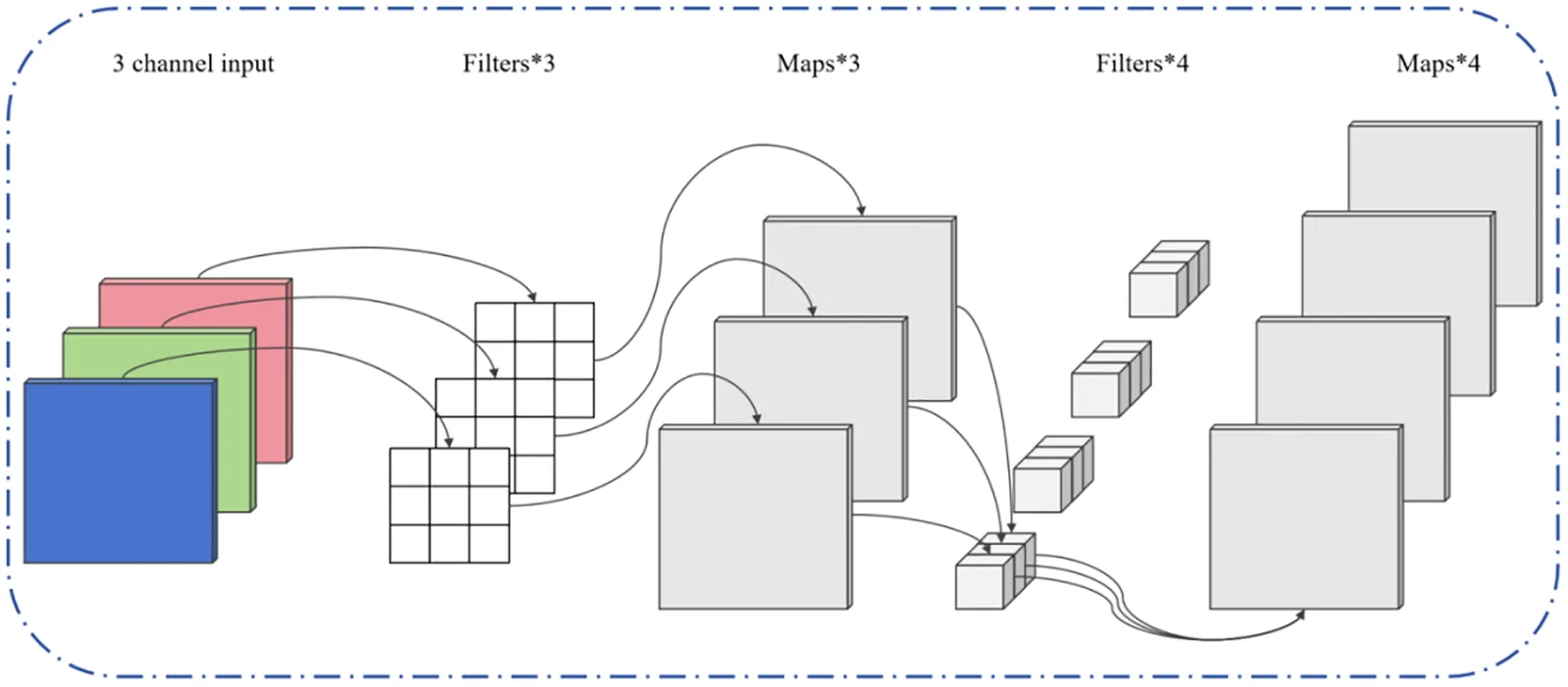
Architecture of the depthwise separable convolution.

In the encoder stage, the conventional convolutional module is restructured using Depthwise Separable Convolution (DS-Conv). As illustrated in **Figure 5**, this operator processes the input feature maps via a decoupled scheme that consists of two independent stages: Depthwise Convolution followed by Pointwise Convolution (Yun et al., 2022). In the Depthwise Convolution stage, convolution is performed independently on each input channel using its corresponding convolution filters/kernels; each kernel operates solely on a single channel to extract spatial features from that channel. This channel-wise processing effectively captures the surface texture of litchi fruits and the linear structures of fruit stems, offering clear advantages for local feature extraction under complex illumination. In the Pointwise Convolution stage, 1×1 convolution filters are applied to the output of the depthwise stage to perform cross-channel feature fusion. By forming a linear combination of spatial features across channels, the pointwise operation integrates and reorganizes multi-dimensional information. For challenging visual patterns in the litchi dataset—such as inter-fruit overlap and occlusion, non-uniform illumination, and blurred boundaries at fruit–stem junctions—this cross-channel fusion enhances the model’s ability to discriminate target boundaries. By combining the outputs of the two stages, the final feature representation is obtained. Because each input channel is convolved independently in the depthwise stage, the number of computations and parameters is substantially reduced relative to standard convolution. In the pointwise stage, the 1×1 kernel size further accelerates computation compared with conventional convolutions. This lightweight design is well-suited to real-time segmentation of litchi images.

Mathematically, the depthwise convolution first operates on the input feature map $F\in R^{D_{F}\times D_{F}\times M}$by performing channel-wise convolution, which is shown in Equation 1:

(1) $${\hat{G}}_{k,l,m}={\sum_{i,j}\hat{K}}_{i,j,m}\cdot F_{k+i-1,l+j-1,m}$$

where $F\;\in \;R^{D_{F}\times D_{F}\times M}$ is the input feature map with spatial size *D_F_* × *D_F_*and M channels; $\hat{K\;}\in \;R^{D_{K}\times D_{K}\times M}$ is the depthwise convolution kernel with kernel size *D_K_* × *D_K_* and M channels; $\hat{G}\;\in \;R^{D_{F}\times D_{F}\times M}$ is the output feature map of the depthwise convolution, maintaining spatial size *D_F_* × *D_F_*and channel number *M*. spatial features through independent channel-wise convolution; subsequently, the pointwise convolution performs cross-channel linear combinations on the output feature maps from the depthwise convolution using 1 × 1 kernels, which is shown in Equation 2:

(2) $$G_{k,l,n}={\sum_{m}\hat{G}}_{k,l,m}\cdot K_{1,1,m,n}$$

Where $\hat{G}\;\in \;R^{D_{F}\times D_{F}\times M}$ is the output feature map from the depthwise convolution; $K\;\in \;R^{1\times 1\times M\times N}\;$is the pointwise convolution kernel mapping M input channels to *N* output channels; $G\;\in \;R^{D_{F}\times D_{F}\times N}$is the output feature map of the pointwise convolution with *N* output channels and spatial size *D_F_* × *D_F_*.

Compared with the computational complexity of standard convolution, *D_K_* × *D_K_* × *M* × *N* × *D_F_* × *D_F_*,the complexity of DS-Conv is *D_K_* × *D_K_* × *M* × *D_F_* × *D_F_* + *M* × *N* × *D_F_* × *D_F_*. The computational reduction ratio is *D_K_*² × *M* × *N* × *D_F_*²/(*D_K_*² × *M* × *D_F_²* + *M* × *N* × *D_F_*²) = *N*/(1 + *N*/*D_K_*²). For *N* = 256 output channels and *D_K_* = 3, this achieves approximately 8.7 × reduction.

### 3.2 Dual cross attention mechanism

The Dual Cross Attention Mechanism (DCA), which combines channel and spatial attentions, has proven effective in feature selection (Ates et al., 2023). In our approach, DCA is positioned strategically within skip connections to address the unique segmentation challenge of slender fruit stems in clustered litchi—namely, capturing their spatial-channel correlations within densely overlapped scenes. This module selectively filters and reinforces informative representations, thereby providing more precise feature selection for multi-scale feature fusion. By strengthening inter-feature associations and enhancing the model’s perception accuracy for the slender morphology of litchi stems and their boundary details, our approach effectively handles the complex geometric characteristics of fruit-stem structures.

**Figure 6:**
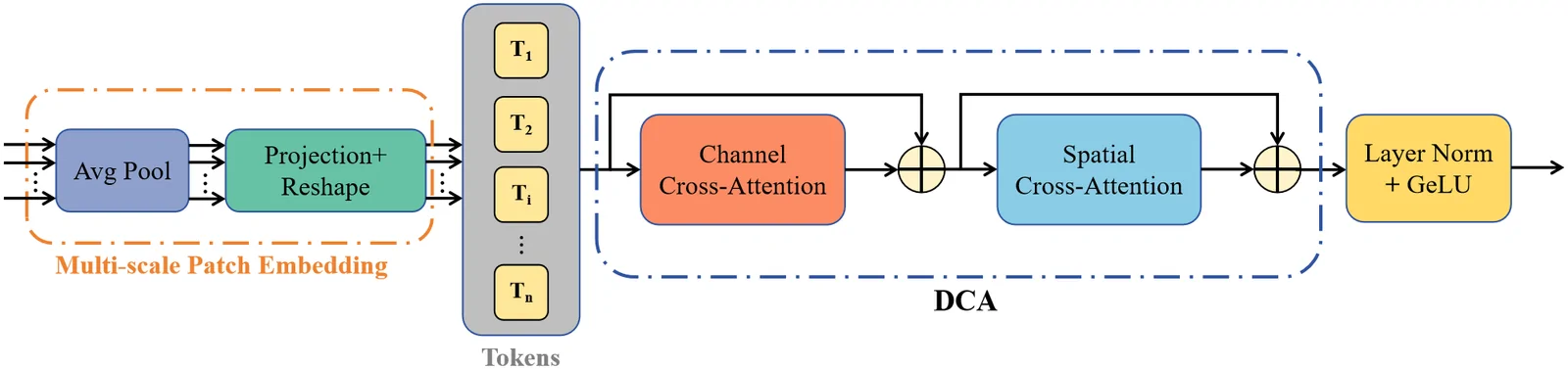
Dual cross-attention module.

As shown in **Figure 6**, the DCA module adopts a hierarchical architecture comprising dual stages and three steps. During the feature extraction and embedding stage, it systematically extracts feature patches from distinct levels of the multi-scale encoder, performs embedding transformations, and generates standardized feature vectors for subsequent cross-attention computations. For the complex geometric characteristics of litchi images—where stems exhibit slender and variable morphologies and fruits cluster densely—this stage effectively captures local details across multiple scales.

At the feature association and fusion stage, the DCA module builds a dual-attention framework consisting of Channel Cross-Attention (CCA) and Spatial Cross-Attention (SCA), progressively modeling long-range dependencies among multi-scale features. This mechanism effectively handles complex illumination and spatial disturbances in litchi images—such as alternating intense direct light and localized shadows, as well as occlusions by branches and leaves—thereby bridging semantic-level discrepancies across scales.

Finally, Layer Normalization and the GeLU activation function are applied to serialize feature tokens for subsequent upsampling operations, enabling effective integration of multi-scale features and propagating refined feature representations to the corresponding decoder layers, which in turn enhances the precision of segmenting clustered litchi fruits and their stems. Among these, the core task of the CCA module (Channel Cross-Attention) is to extract global dependencies among channels, and its main formulation is shown in Equations 3, 4.

where *T_i_*is the input feature map encompassing multi-dimensional features from input images; Tc represents the channel-focused features for generating key and value matrices; *Q_i_*, *K*, *V* are the query, key, and value matrices generated through 1*D* depthwise convolution; *C* denotes the channel dimension; *C_c_* is the channel dimension for scaling normalization; $1/\sqrt{c_{c}}$ is the scaling factor preventing gradient vanishing in softmax computation. The output of the CCA module is a weighted sum of the values, where the weights are determined by the similarity between the queries and the keys. Finally, a DS-Conv is applied to the cross-attention output before feeding it into the SCA module.

The SCA module (Spatial Cross-Attention) focuses on capturing spatial dependencies, which can be formulated as shown in Equations 5, 6.

where ${\bar{T}}_{c}$ refers to the concatenated tokens from the CCA module, used as queries and keys; ${\bar{T}}_{i}$ represents each token used as a value in the attention calculations; $Q,\;K\;\in \;R^{P\times C}$ are the query and key matrices generated from concatenated CCA tokens; $V_{i}\;\in \;R^{P\times C}$is the value matrix generated from individual input tokens; *P* and *C* denote the spatial and channel dimensions, respectively; *d_k_*is the dimensionality of the key vector for each head; *h_c_* denotes the number of heads in the multi-head attention mechanism; *C_c_* is the overall feature dimension uniformly partitioned into heads;

(3) $$Q_{i}=DConv1D_{Q_{i}}\;(T_{i})\;,\;\;K=DConv1D_{K}\;(T_{c})\;,\;\;V=DConv1D_{V}(T_{c})$$

(4) $$CCA(Q_{i},K,V)=Soft\max(\frac{Q_{i}^{T}K}{\sqrt{C_{c}}})V^{T}$$

(5) $$Q=DConv1D_{Q}(\;{\bar{T}}_{c}),K=DConv1D_{K}(\;{\bar{T}}_{c})\;,V_{i}=DConv1D_{V_{i}}(\;{\bar{T}}_{i})$$

(6) $$SCA(Q,K,V_{i})=Soft\max(\frac{QK^{T}}{\sqrt{d_{k}}})V_{i}$$

$1/\sqrt{d_{k}}$is the scaling factor preventing the softmax function from entering the saturation region. This setup allows the SCA module to effectively capture spatial dependencies. Subsequently, a DS-Conv is applied to the output of SCA to obtain the final output of DCA.

### 3.3 Loss function and model training strategy

In the collected litchi images, the unified foreground mask defined in Section 2.2 is internally heterogeneous in pixel composition. Fruit-dominated areas account for the majority of foreground pixels, whereas the slender fruit–stem connection regions occupy a much smaller proportion. During training, this within-foreground pixel imbalance induces biased learning toward fruit-dominated areas, which markedly weakens the model’s representation of slender connective structures and degrades segmentation continuity along fruit–stem connections.

In addition, the litchi stem exhibits a slender, strip-like topology with a high aspect ratio (typically 15:1–25:1) and complex curvature. Under cluttered backgrounds, this often leads to discontinuous segmentation and fragmented shapes.

The conventional Binary Cross-Entropy Loss (BCE-Loss) performs independent per-pixel classification and lacks explicit constraints on object geometry and spatial continuity (Kim, 2016), making it difficult to preserve boundary integrity for slender stem segmentation. For the binary segmentation task adopted in this study, these challenges are particularly pronounced along morphologically distinct yet topologically connected subregions within the unified foreground mask. Therefore, we design a composite loss that integrates pixel-level classification with geometric shape constraints, termed the Binary Cross-Entropy–Level Set Mixed Loss (BL-Loss). This loss combines the classification accuracy of BCE-Loss with the geometric regularization of Level Set Loss (LS-Loss). Specifically, LS-Loss introduces distance-transform and curvature constraints to effectively maintain the topological continuity and shape integrity of the stem region (Wang et al., 2018), enabling precise and continuous segmentation of litchi stems. The formulations of the composite loss, BCE-Loss, and LS-Loss are presented below which are shown in Equations 7 , 8 and 9:

(7) $$L_{BL}=\lambda_{BCE}\cdot L_{BCE}(X)+\lambda_{LS}\cdot L_{LS}(X)$$

(8) $$L_{BCE}(X)=-\frac{1}{N}\sum_{i=1}^{N}\left[y_{i}\log({\hat{y}}_{i})+(1-y_{i})\log(1-{\hat{y}}_{i})\right]$$

(9) $$L_{LS}(X)=\alpha \int {\left(H(\varphi -y)\right)}^{2}dx+\beta \int \delta (\varphi )\cdot ∥\nabla \varphi ∥\;dx(X+1)$$

where *L_BCE_* denotes the BCE-Loss, *L_LS_* denotes the LS-Loss, *λ_BCE_* and *λ_LS_* are the balancing weights for the two loss functions. Here, *X* represents the input image on which the loss functions are calculated; *N* is the total number of pixels in the image; *y_i_* and ${\hat{y}}_{i}$denote the ground-truth label and the predicted probability of the *i*-th pixel, respectively; H(φ)is a differentiable Heaviside function used to map the predicted logits to [0,1]; δ(φ)is a differentiable Dirac Delta function used to emphasize the zero level-set boundary; ∥∇φ∥is the gradient magnitude that quantifies boundary strength; α and β are the weighting coefficients for the region discrepancy term and the boundary smoothing term, respectively.

Within LS-Loss, the region discrepancy term $\alpha \int {\left(H(\phi -y)\right)}^{2}$ leverages the global constraint property of the Heaviside function, which not only focuses on per-pixel classification accuracy but, more importantly, enforces consistency of the overall region shape, thereby preserving the intact morphology of litchi clusters. In contrast, the boundary smoothing term β∫δ(φ)·∥∇φ∥ dximposes constraints in the vicinity of the zero level set via the Dirac Delta function, and intrinsically tends to maintain the topological integrity of connected structures. This exerts a regularization effect on the continuous segmentation of the slender stem and effectively suppresses segmentation fragmentation caused by illumination changes and background clutter.

To prevent complex constraints from interfering with the learning of basic features at the early stage of training, a phased training strategy is adopted: during the initial 200 epochs, only the BCE-Loss is used for coarse segmentation learning; in the subsequent 200 epochs, the composite loss is employed with λ_BCE = 1.0 and λ_LS = 0.5. The relatively smaller weight for LS-Loss reflects its role as a geometric refinement mechanism: since the level-set constraint is introduced after basic features have been sufficiently learned, a balanced weighting ensures that shape regularization enhances rather than dominates the learning process, preventing training instability from imposing strong topology constraints during the refinement phase. This strategy effectively mitigates the pixel imbalance problem in litchi-cluster segmentation and markedly improves the segmentation continuity of slender stems as well as the overall segmentation quality.

## 4 Results and analysis

### 4.1 Experimental environment

Model training and picking-point localization experiments were conducted on two separate servers. The server used for segmentation model training was equipped with a Central Processing Unit (CPU), specifically an Intel(R) Xeon(R) Gold 6330, with a base frequency of 2.00 GHz and a maximum frequency of 3.10 GHz in a system with dual processors; the Graphics Processing Unit (GPU) was an NVIDIA GeForce RTX 4090 with 24 GB of video memory. The server used for picking-point localization was configured with an AMD Ryzen Threadripper 3960X (24-core, 3.80 GHz) CPU; the GPU was an NVIDIA GeForce RTX 2080 with 8 GB of video memory. The software environment included a 64-bit Ubuntu operating system, PyCharm, the PyTorch deep learning framework, and the Python programming language (Python 3.9).

### 4.2 Evaluation metrics

To evaluate the proposed method in terms of object detection and segmentation, the following metrics were adopted: Mean Intersection over Union (*mIoU*, %) and Mean Pixel Accuracy (*mPA*, %) were selected as accuracy indicators of the segmentation performance for clustered litchi fruits and stems (Long et al., 2015); *Precision* (%), *Recall* (%), and *F1-score* (%) were employed to quantify the detection performance; and Frames Per Second (*FPS*) was used to assess the model’s real-time processing capability. Intersection over Union (*IoU*) is used to quantify the overlap between the predicted region and the ground-truth region, and is calculated as the ratio of the intersection area to the union area. *mIoU* is the average *IoU* across all classes, which can be caluated by Equation 10.

(10) $$mIoU=\frac{1}{N}\cdot \sum_{i=1}^{N}\frac{p_{ii}}{\sum_{j=1}^{N}p_{ij}+\sum_{j\neq i}p_{ji}}$$

where the denominator is the union of the number of pixels labeled as class *i* and those misclassified as class *j*. Here, *P_ii_* denotes the number of pixels correctly classified as class *i*; *P_ij_* represents the number of pixels labeled as class *i* but misclassified as class *j*; while *P_ji_* indicates the pixels labeled as class *j* but misclassified as class *i*. A higher *mIoU* indicates stronger model performance in segmentation tasks. *mPA* denotes the average, over all classes, of the percentage of pixels that are correctly classified in a segmentation map. Let *p_ij_* denote the number of pixels from class *j* that are classified as class *i*. Then $\sum_{j=1}^{N}p_{ij}$represents the total number of pixels predicted as class *i*, *i.e*., all pixels assigned to class *i*. Accordingly, *mPA* is defined as follows (Equation 11):

(11) $$mPA=\;\frac{1}{N}\sum_{i=1}^{N}\frac{p_{ii}}{\sum_{j=1}^{N}p_{ij}}$$

*Precision*, *Recall*, and *F1-score* quantify, respectively, the detection accuracy, detection completeness, and overall performance of the model; the formulas are defined as follows (Equations 12–14):

(12) $$Precison=\;\frac{TP}{TP+FP}$$

(13) Recall= TPTP+FN

(14) F1−score= 2Precison·RecallPrecison+Recall

Here, *TP* denotes the number of true positive detections of litchi fruit stems; *FP* denotes the number of false positive detections (misdetections) of litchi fruit stems; and *FN* denotes the number of false negatives (missed detections) of litchi fruit stems. *FPS* indicates the number of image frames the model can process per unit time and is used to measure inference speed. The *FPS* is defined as follows (Equation 15):

(15) FPS=1/T

where *T* denotes the average time required by the model to process a single image frame (in seconds). A higher *FPS* indicates that the model achieves faster processing speed in inference tasks and better real-time performance.

### 4.3 Segmentation results and analysis for litchi fruit clusters

#### 4.3.1 Comparative performance of different models

To validate the effectiveness of the proposed DSD-UNet, comprehensive comparative experiments on litchi fruit cluster segmentation were conducted on the same dataset against ten representative methods, including classical semantic segmentation architectures such as the original U-Net (Ronneberger et al., 2015), PSPNet with a pyramid pooling module (Zhu et al., 2021), the fully convolutional network FCN (Long et al., 2015), and HRNet focused on maintaining high-resolution feature representations (Liu et al., 2024); Transformer-based approaches including Segformer (Zhang et al., 2023), CMTFNet that fuses CNN and multiscale Transformer architectures (Wu et al., 2023), and MaSSFormer (Xie et al., 2025), a recently proposed method designed for high-resolution semantic segmentation; lightweight segmentation networks including Fast-SCNN (Liu J. et al., 2023) and PIDNet-Lite (Liu et al., 2025); and YOLO11n-seg (Khanam and Hussain, 2024), a lightweight YOLO-based instance segmentation model widely used in real-time vision pipelines.

All models were trained on the training set and evaluated on the test set. To ensure fairness and comparability, all baselines adopted identical training configurations: learning rate (lr=0.001), batch size (8), maximum training epochs (400), and the Adam optimizer for parameter updates. The comparative segmentation results for litchi clusters under different methods are presented in **Table 2**.

**Table 2:** Comparative performance of different models in detection and segmentation on the test set.

Model	*mIoU* (%)	*Precision* (%)	*Recall* (%)	*F1-score* (%)	*mPA* (%)	*FPS*	*Model size* (M)
**PSPNet**	86.34 (-1.98)	89.86 (-4.19)	**95.66 (+1.89)**	92.67 (-0.94)	98.22 (-0.35)	15.20 (+8.75)	42.12 (+28.72)
**Segformer**	80.27 (-8.05)	92.78 (-1.27)	85.60 (-8.17)	88.96 (-4.65)	97.58 (-0.99)	5.10 (-1.35)	13.75 (+0.35)
**FCN**	81.67 (-6.65)	91.41 (-2.64)	88.54 (-5.23)	89.89 (-3.72)	97.69 (-0.88)	6.21 (-0.24)	11.74 (-1.66)
**CMTFNet**	87.80 (-0.52)	93.08 (-0.97)	93.92 (+0.15)	93.50 (-0.11)	98.46 (-0.11)	3.98 (-2.47)	30.19 (+16.79)
**HRNet**	82.78 (-5.54)	90.21 (-3.84)	90.95 (-2.82)	90.58 (-3.03)	97.78 (-0.79)	4.67 (-1.78)	29.81 (+16.41)
**Fast-SCNN**	84.94 (-3.38)	90.51 (-3.54)	93.25 (-0.52)	91.86 (-1.75)	98.06 (-0.51)	8.85 (+2.40)	4.86 (-8.54)
**PIDNet-Lite**	81.93 (-6.39)	91.58 (-2.47)	88.61 (-5.16)	90.07 (-3.54)	97.70 (-0.87)	5.32 (-1.13)	3.27 (-10.13)
**MaSSFormer**	85.54 (-2.78)	93.11 (-0.94)	91.32 (-2.45)	92.21 (-1.40)	98.19 (-0.38)	12.93 (+6.48)	13.50 (+0.10)
**YOLO11n-seg**	78.28 (-10.04)	91.19 (-2.86)	85.30 (-8.47)	86.85 (-6.76)	97.14 (-1.43)	11.96 (+5.51)	**2.84 (-10.56)**
**Original_U-Net**	88.32	94.05	93.77	93.61	98.57	6.45	13.40
**Our model**	**91.40 (+3.08)**	**95.73 (+1.68)**	95.37 (+1.60)	**95.42 (+1.81)**	**98.95 (+0.38)**	**15.41 (+8.96)**	5.16 (-8.24)

The bold values represent the optimal value among all the comparison indicators.

As shown in **Table 2**, the comparative performance results of litchi fruit cluster segmentation under different methods are presented (bold numbers indicate the best performance, underlined entries denote the baseline model, and values in parentheses represent the performance differences relative to the baseline model). The results demonstrate that the proposed model achieves optimal performance across key evaluation metrics including segmentation accuracy and inference efficiency, with *mIoU* of 91.40%, *Precision* of 95.73%, *Recall* of 95.37%, *F1-score* of 95.42%, *mPA* of 98.95%, inference speed of 15.41 *FPS*, and a model size of only 5.16M.

Compared with the baseline methods, the proposed model improves *mIoU* by 3.08 percentage points, increases *FPS* by 8.96, and reduces model size to 38.5% of Original_U-Net. Relative to PSPNet and HRNet, it achieves improvements of 5.06 and 8.62 percentage points in *mIoU* respectively, and outperforms HRNet by 10.74 *FPS*. Compared with recent advanced methods, the proposed model surpasses CMTFNet by 3.60 percentage points in *mIoU* with a significant *FPS* increase of 11.43; relative to the Transformer-based Segformer, it achieves 11.13 percentage points improvement in *mIoU* and 10.31 *FPS* enhancement; it also outperforms MaSSFormer by 5.86 percentage points in *mIoU*. Among lightweight methods, the proposed model demonstrates superior segmentation accuracy and inference speed compared to Fast-SCNN and PIDNet-Lite. Compared with YOLO11n-seg, the proposed model surpasses it by 13.12 percentage points in *mIoU* and outperforms it by 3.45 *FPS*. Although YOLO11n-seg has the smallest parameter count (2.84M) among all compared methods, its segmentation accuracy on the unified fruit-with-stem task remains substantially lower than that of DSD-UNet. These experimental results verify the effectiveness and advancement of the proposed method, achieving a good balance between segmentation performance, real-time processing capability, and model compactness.

**Figure 7:**
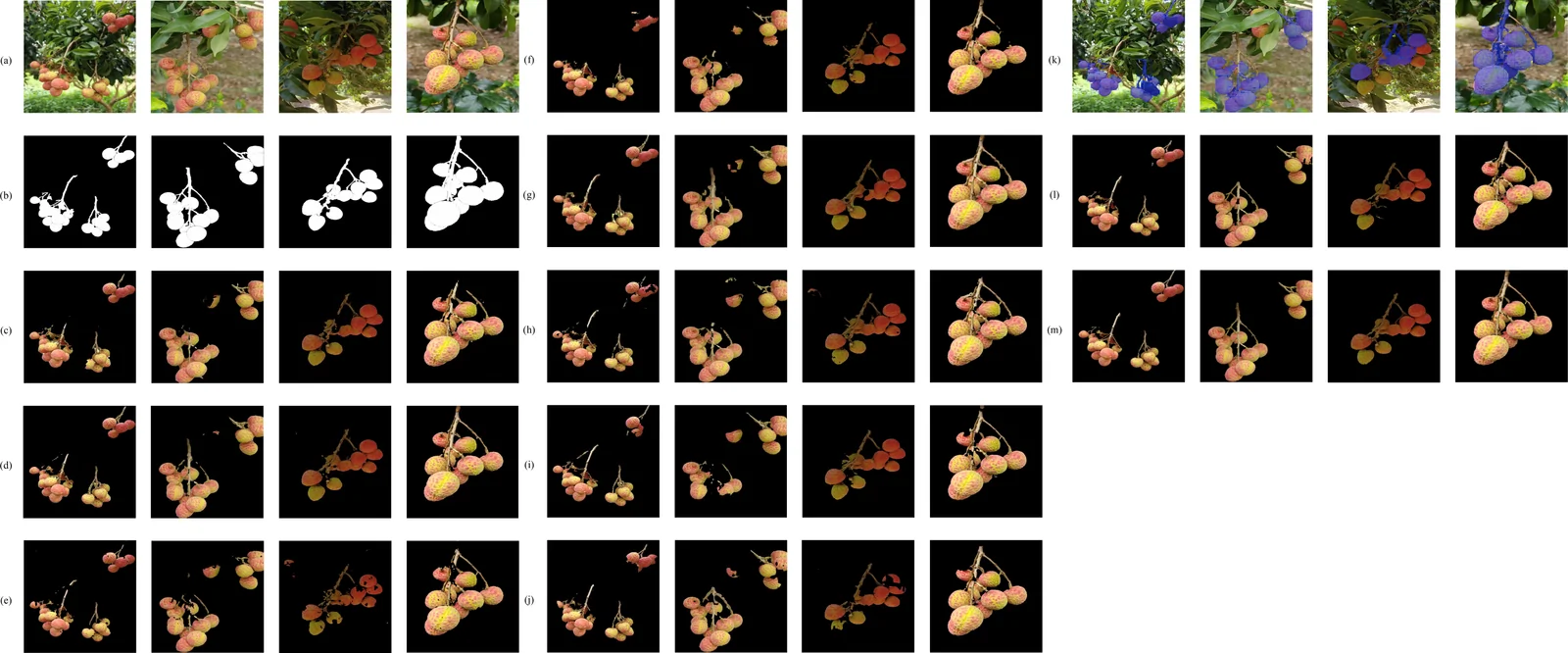
Segmentation results of litchi clusters using different models: **(a)** Original images **(b)** Real area; **(c)** HRNet; **(d)** CMTFNet; **(e)** FCN; **(f)** Segformer; **(g)** PSPNet; **(h)** Fast-SCNN; **(i)** PIDNet-Lite; **(j)** MaSSFormer; **(k)** YOLO11n-seg; **(l)** U-Net; **(m)** our model.

As shown in **Figure 7**, the performance of different models in segmenting target regions of litchi clusters varies markedly. Overall, FCN and Segformer deliver relatively poor detail segmentation, particularly when faced with illumination-induced shadows, complex segmentation objects, or background distractors, where undersegmentation and mis-segmentation tend to occur. PSPNet and Original U-Net provide stable overall segmentation results but exhibit certain limitations in edge precision and detail processing, most notably in maintaining boundary continuity and segmentation completeness for fruit stem regions where further improvements are needed.

Among recent advanced methods, CMTFNet demonstrates good performance in multi-scale feature fusion and overall segmentation capability, but still shows gaps compared to the proposed method in handling complex-shaped targets containing fine fruit stems and maintaining segmentation consistency. For Transformer-based approaches, MaSSFormer, despite its optimization for high-resolution segmentation, exhibits inferior detail processing performance in complex litchi clusters. Lightweight methods such as Fast-SCNN and PIDNet-Lite, though featuring reduced model sizes, suffer from limited capability in capturing fine-grained features due to their network architecture constraints; consequently, they tend to produce undersegmentation and boundary fragmentation when dealing with multi-target aggregation and detailed fruit stem delineation, failing to achieve an effective balance between accuracy and efficiency. As shown in **Figure 7k**, YOLO11n-seg provides instance-level detections with competitive inference efficiency, but tends to produce incomplete masks and boundary discontinuities along slender fruit–stem connections in densely clustered regions, which is consistent with its lower *Recall* and *mIoU* reported in **Table 2**.

In contrast, the proposed DSD-UNet achieves the best segmentation performance across all scenarios; apart from minor flaws in segmenting the extremely small target fruit on the far left in the first image and minor undersegmentation at the central fruit stem in the fourth image, it significantly reduces both undersegmentation and mis-segmentation relative to the other models, demonstrating superior performance in precise fruit boundary segmentation and maintaining fruit stem continuity. In summary, DSD-UNet exhibits superior performance for segmentation tasks involving cluttered backgrounds and complex segmentation targets.

#### 4.3.2 Ablation study

To elucidate the specific contributions of each improvement, a systematic ablation study was conducted on three core components— the feature extraction network, the attention mechanism module, and the loss function—with the results summarized in **Table 3**. Quantitative analysis showed that the DCA attention module contributed most significantly to performance gains: relative to the baseline U-Net, integrating DCA alone improved *mIoU* by 1.57 percentage points (from 88.32% to 89.89%), validating the effectiveness of this attention mechanism in feature enhancement. Replacing the original feature extraction network with Ds-Convs achieved substantial model compression: the parameter count was reduced to 38.5% of Original U-Net (from 13.40M to 5.16M, a 61.5% reduction), while segmentation accuracy remained comparable. When integrated with DCA and BL-Loss, the full model achieves 3.08 percentage points higher mIoU than the baseline while maintaining the 61.5% parameter reduction.

**Table 3:** Comparison of ablation experiment results for the U-Net-based semantic segmentation model.

DS-Conv	DCA	BL-Loss	*mIoU* (%)	*mPA* (%)	*Precision* (%)	*Recall* (%)	*F1-score* (%)	*Model size* (M)
–	–	–	88.32	98.57	94.05	93.77	93.61	13.40
✓	–	–	87.47	98.45	94.08	92.72	93.13	5.06
–	✓	–	89.89	98.77	95.44	94.05	94.45	13.49
–	–	✓	88.68	98.63	94.62	93.59	93.80	13.40
✓	✓	–	89.74	98.75	95.78	93.55	94.37	5.16
–	✓	✓	91.09	98.92	95.52	95.26	95.23	13.49
✓	–	✓	88.09	98.55	94.16	93.32	93.50	5.06
✓	✓	✓	**91.40**	**98.95**	**95.73**	**95.37**	**95.42**	5.16

The bold values represent the optimal value among all the comparison indicators.

The synergistic effect between the BL-Loss and the DCA attention module was particularly notable: their combination increased *mIoU* to 91.09%, an improvement of 2.77 percentage points over the baseline, which markedly exceeds the simple sum of their individual contributions, demonstrating a strong synergistic effect between loss optimization and attention (Yeung et al., 2022). When all modules act in concert, the model attains its optimal configuration, achieving an *mIoU* of 91.40% and an *F1-score* of 95.42%, while retaining a lightweight profile with only 5.16M parameters. These ablation results substantiate the effectiveness of each improvement and its synergistic optimization mechanism, providing empirical support for the soundness of the model design.

**Figure 8:**
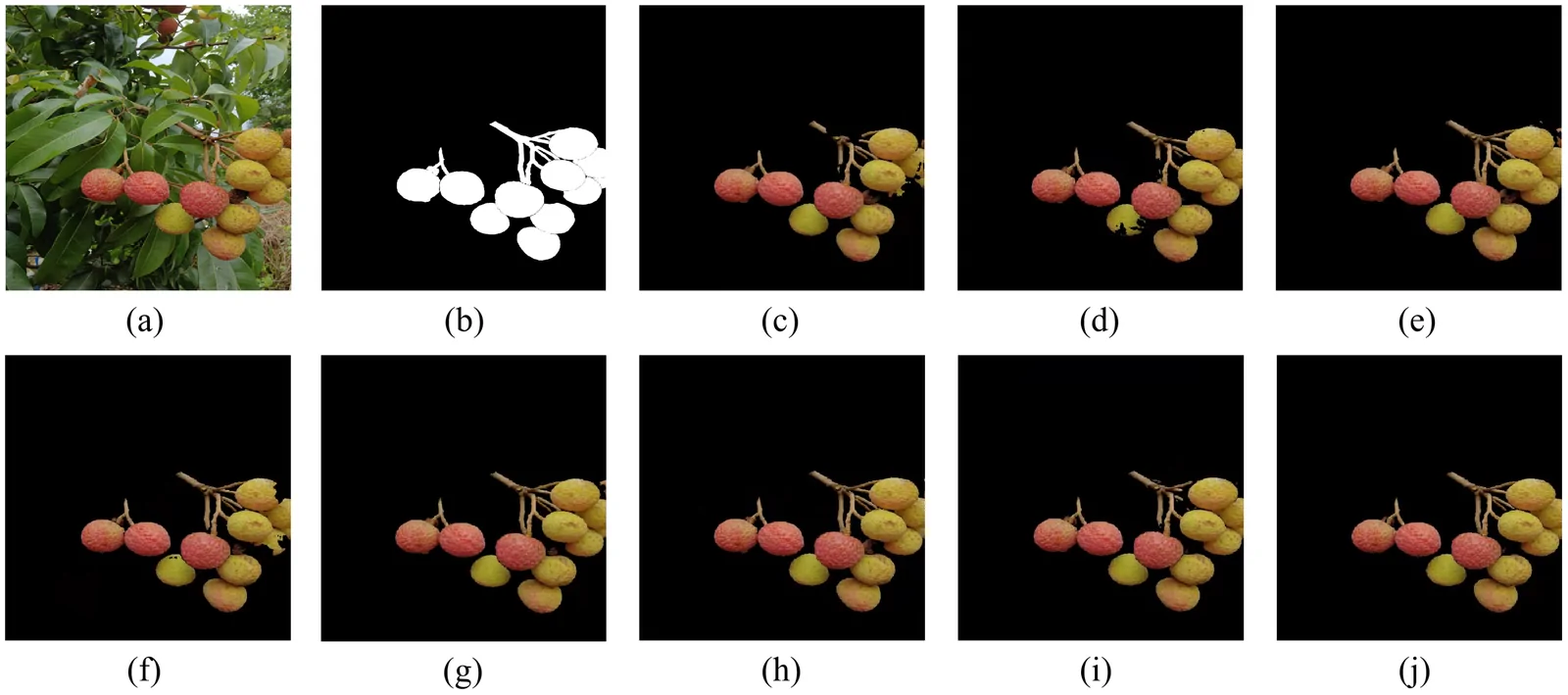
Ablation study visualization results: **(a)** Original image **(b)** Real area; **(c)** Baseline; **(d)**+DS-Conv; **(e)** +DCA; **(f)** +BL-Loss; **(g)** +DS-Conv+DCA; **(h)** +DCA+BL-Loss; **(i)**+DS-Conv+BL-Loss; **(j)** our model.

As illustrated in **Figure 8**, the visualization results from ablation experiments intuitively demonstrate the specific impact of different module combinations on segmentation quality, corroborating the quantitative analysis findings. The visual effects observed in **Figure 8d** reveal that depthwise separable convolution achieves model lightweighting while maintaining segmentation quality comparable to the baseline model, validating the effectiveness of parameter compression strategies. However, minor deficiencies remain in processing fine structures such as fruit stem connections, aligning with the slight decrease in its *mIoU* performance indicated by quantitative results. **Figure 8e** demonstrates the application effects of the dual cross-attention mechanism, showing that DCA significantly improves segmentation continuity for elongated structures like fruit stems by enhancing feature representation capability and reducing discontinuity phenomena, which corresponds to the 1.57 percentage point improvement in *mIoU*. **Figure 8f** displays the improvement effects of BL-Loss in boundary smoothness and target completeness, with segmentation contours more closely fitting true boundaries, visually validating the effectiveness of BL-Loss in optimizing segmentation boundary quality.

Comparing the segmentation results of **Figures 8h–j**, clear improvements in visual quality brought by module combinations can be observed. The combination configuration of DCA and BL-Loss in **Figure 8h** demonstrates particularly outstanding performance in maintaining fruit stem topological integrity, with this visual improvement effect being highly consistent with the 2.77 percentage point *mIoU* enhancement shown in quantitative analysis. The final complete configuration (**Figure 8j**) exhibits optimal visual segmentation effects under complex background and illumination variation conditions. The visualization results validate the quantitative analysis conclusions from a visual perspective, confirming that the synergistic effects of various improvement modules across different aspects ultimately achieve comprehensive enhancement in segmentation performance.

#### 4.3.3 Post-processing

To further enhance the segmentation performance of DSD-UNet, the Conditional Random Fields (CRF) is employed as post-processing on the network outputs (Mahmood and Durr, 2018) of litchi stems. Specifically, the Gaussian kernel was configured with a spatial distance parameter (sxy = 3) and a compatibility weight (compat = 3) to enforce local spatial consistency within neighborhoods; the bilateral kernel was set with (sxy = 20), color similarity (srgb = 4), and (compat = 10) to jointly account for spatial position and color-feature similarity. **Table 4** reports the performance comparison of DSD-UNet with and without CRF post-processing. After CRF-based post-processing, the primary metric *mIoU* increased from 91.40% to 91.80%, an improvement of 0.40 percentage points. Meanwhile, other metrics, including *Precision*, *Recall*, *F1-score*, and *mPA*, exhibited modest improvements across all metrics, corroborating the effectiveness of CRF post-processing in improving litchi stem segmentation accuracy.

**Table 4:** Segmentation metrics of DSD-UNet with and without CRF post-processing.

Model	*mIoU (%)*	*Precision (%)*	*Recall (%)*	*F1-score (%)*	*mPA (%)*
DSD-UNet	91.40	95.73	95.37	95.42	98.95
**DSD-UNet-CRF**	**91.80**	**95.93**	**95.62**	**95.64**	**98.99**

The bold values represent the optimal value among all the comparison indicators.

#### 4.3.4 Failure case analysis

**Figure 9:**
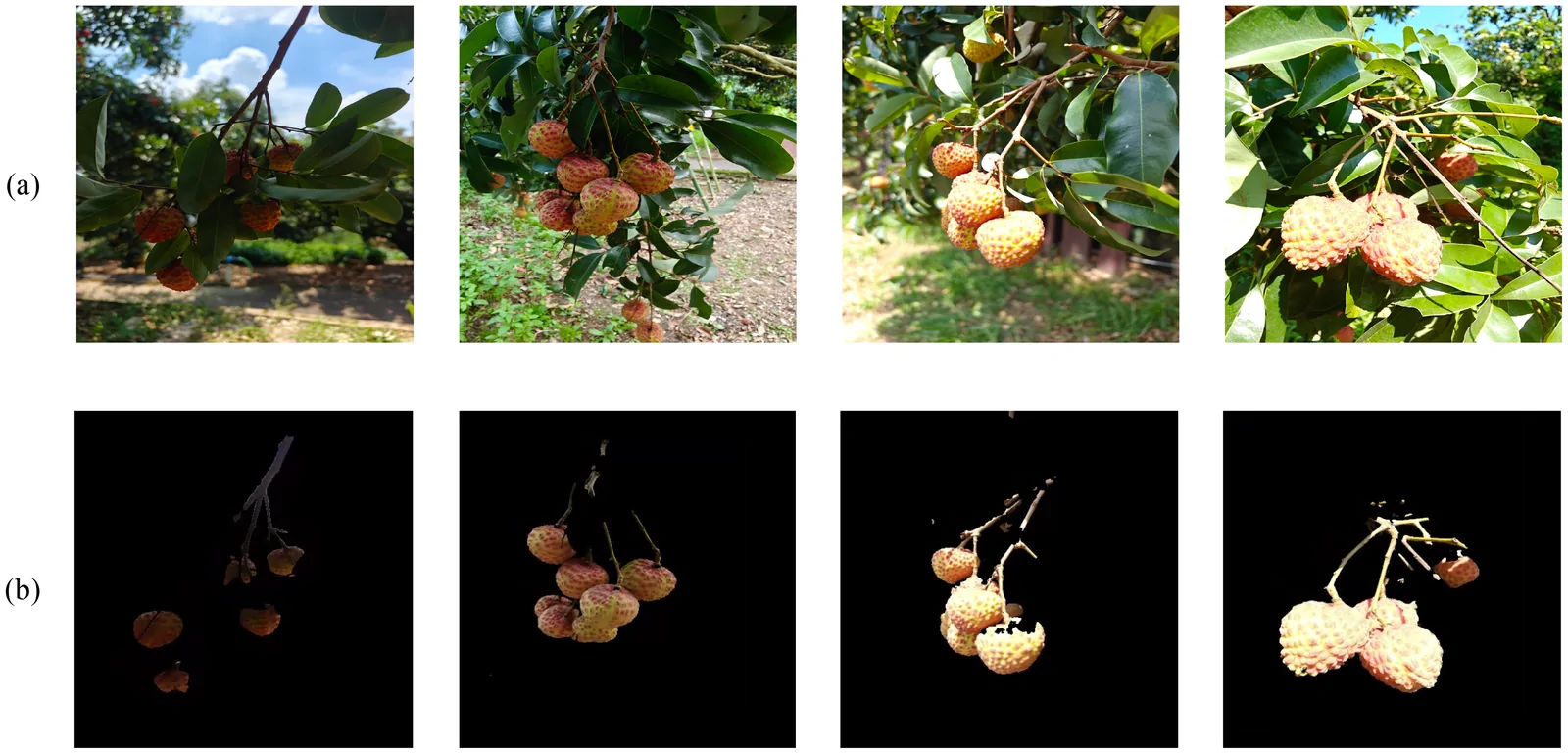
Failure case analysis under extreme environmental conditions: **(a)** original images with challenging scenarios; **(b)** corresponding segmentation results showing model limitations.

Although the proposed model demonstrates superior performance on standard test sets, it still exhibits limitations under extreme environmental conditions. **Figure 9** presents four typical failure cases, and analyzing these scenarios helps understand model boundaries and guide improvement directions.

Among these challenging scenarios, the most complex case involves simultaneous occurrence of severe leaf occlusion, dense fruit overlap, and low-light conditions. The segmentation results reveal significant target omission and boundary incompleteness issues. Low illumination weakens feature extraction effectiveness, while severe occlusion restricts available information, preventing the dual cross-attention mechanism from establishing effective long-range dependencies. Another prominent challenge emerges in high-density distribution scenarios where densely arranged litchi fruits interfere with complex backgrounds. The model struggles to accurately distinguish individual boundaries, resulting in fruit adhesion and shape distortion. The devastating effects of lighting extremes become particularly evident in the remaining cases, where overexposure and uneven illumination dominate.

Overexposure causes severe image information loss, and lighting non-uniformity exacerbates segmentation difficulty. In strong light regions, boundaries between fruits and background disappear, while detail information is significantly compressed in shadow areas. Although the hybrid loss function improves boundary quality, it remains insufficient when facing severe information loss.

Failure case analysis reveals three main limitations: (1) limited lighting adaptability; (2) insufficient shape inference under severe occlusion; (3) inadequate individual separation capability in dense scenarios. Future improvements can be pursued in the following directions: introducing illumination-invariant feature extraction methods to enhance robustness; integrating multi-modal information (depth, spectral data, etc.) to improve recognition capability in occluded scenarios; developing specialized dense target separation mechanisms to enhance segmentation accuracy in high-density scenes.

### 4.4 Qualitative analysis of picking point localization based on segmentation results

To verify the practical value of segmentation results for downstream tasks, we designed a picking point localization experiment to evaluate the support provided by the DSD-UNet segmentation model for litchi harvesting robot vision systems. This experiment aims to validate whether accurate litchi segmentation results can effectively improve picking point localization accuracy, thereby providing more reliable technical support for actual robotic harvesting operations. Under various weather and lighting conditions, we compared three picking-point localization schemes based on the YOLOv8n-pose keypoint detection network (Wang J. et al., 2024). The Direct Picking-point Localization (DPL) method uses original images as input. The Fast-SCNN-Guided Picking-point Localization (FSGPL) approach uses Fast-SCNN as the lightweight segmentation representative; among the lightweight semantic segmentation baselines in **Table 2**, Fast-SCNN achieves the highest mIoU (84.94%) and has a model size (4.86M) closest to that of DSD-UNet (5.16M). Its segmentation results are fed into the YOLOv8n-pose network. The Segmentation-Guided Picking-point Localization (SGPL) approach feeds the segmentation results produced by DSD-UNet into the same pose network. The three methods employ an identical localization model and differ only in the form of input data, ensuring fair comparison. The mean calculation time reported in **Table 5** refers to the picking-point localization stage based on the prepared segmentation inputs, and does not include the inference time of the segmentation models.

**Table 5:** Quantitative comparison of DPL, FSGPL and SGPL for picking-point localization.

Method	Images	Targets	Successful locations	Failed locations	Success rate(%)	Mean calculation time(s)
DPL	100	132	55	77	41.67	**48.2×10^-3^**
FSGPL	100	132	94	38	71.21	55.4×10^-3^
SGPL	100	132	119	13	90.15	55.2×10^-3^

The bold values represent the optimal value among all the comparison indicators.

To ensure the objectivity of experimental results, we manually annotated optimal picking points at the fruit stem connections of each litchi cluster under the guidance of agricultural experts. Based on these annotated points, we defined 35 × 20 pixel rectangular regions centered on the picking points as “optimal picking regions.” These dimensions were determined based on the grasping precision requirements of actual mechanical grippers to ensure that robotic grasping within this range would neither damage the fruit nor fail to successfully separate the entire litchi cluster. Localization success or failure is determined based on whether the predicted picking point falls within the manually defined optimal picking region, which serves as the unified standard for assessing localization accuracy and conducting performance evaluation.

**Figure 10:**
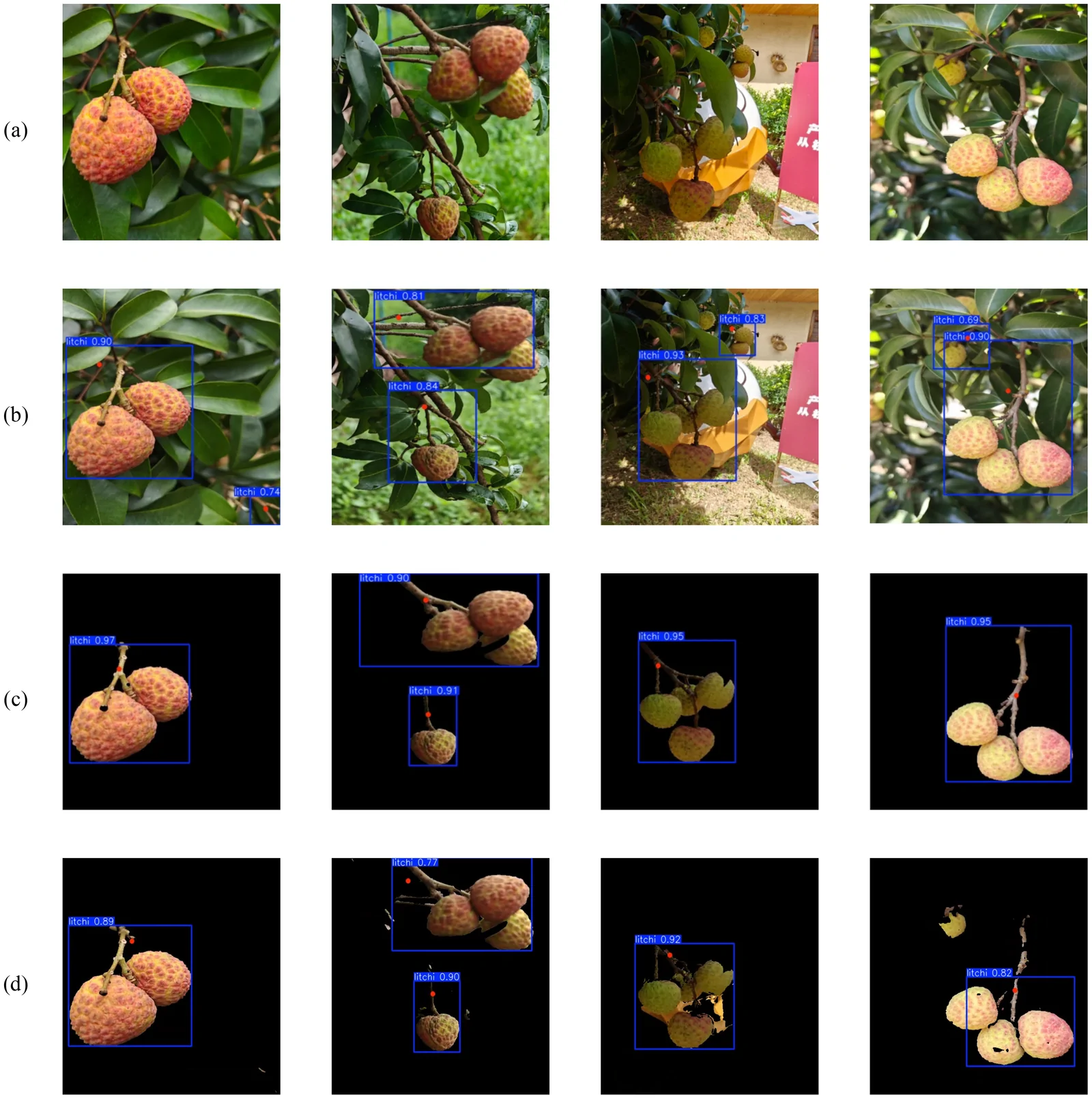
Comparative visualization of picking-point localization methods under various scenes: **(a)** Original image; **(b)** DPL; **(c)** FSGPL; **(d)** SGPL.

As shown in **Figure 10**, for various natural scenes, each case presents the original input and the localization results obtained by DPL, FSGPL, and SGPL. The DPL approach is notably prone to false positives and misses, with predicted points frequently deviating from the actual fruit-bearing stems and susceptible to interference from leaves or complex backgrounds, resulting in limited overall accuracy. FSGPL benefits from Fast-SCNN segmentation priors and achieves visibly improved point concentration relative to DPL; however, under-segmentation and mis-segmentation of fruit–stem structures still markedly degrade localization accuracy, as exemplified in the second and third scenes of **Figure 10**. In these cases, non-target pixels introduced by mis-segmentation can displace the predicted picking point away from the true fruit–stem junction. When slender connective regions are fragmented or omitted, the pose model may still return a detectable keypoint, but not at the optimal picking location, because the corresponding stem region is absent from the segmentation mask.

In contrast, SGPL—leveraging DSD-UNet segmentation—more effectively suppresses background clutter, preserves connective fruit–stem structures, and places localization points closer to the optimal picking locations under varying conditions.

Further quantitative results are summarized in **Table 5**. Among 132 targets in 100 test images, SGPL successfully localized 119 targets (a success rate of 90.15%) with a mean calculation time of 55.2 ms, FSGPL localized 94 targets (a success rate of 71.21%) with 55.4 ms, and DPL localized 55 targets (41.67% success rate) with 48.2 ms. Although DPL retains the shortest mean calculation time, the two segmentation-guided methods require comparable localization time (~55 ms) while achieving substantially higher success rates. Compared with FSGPL, SGPL improves the success rate by 18.94 percentage points, indicating that the downstream localization benefit depends on the quality of the segmentation prior rather than on the use of segmentation alone.

Analysis reveals that the core improvement in SGPL performance lies in the provision of structural priors and background suppression by the segmentation model, which enables effective filtering of non–fruit-bearing stems, interfering foliage, and complex textures, markedly reducing the probability of false positives and missing detections. Additionally, DSD-UNet exhibits exceptional capability in topological representation and boundary delineation of slender structures such as fruit stems, further increasing both the concentration and accuracy of the localized picking points and effectively mitigating positional deviations.

The intermediate performance of FSGPL is consistent with the segmentation results in **Table 2**, where Fast-SCNN—selected as the lightweight representative owing to its leading *mIoU* among lightweight baselines and a comparable parameter scale to DSD-UNet—still achieves a lower *mIoU* (84.94%) than DSD-UNet (91.40%). This indicates that the advantage of SGPL is associated with the higher-quality unified fruit-with-stem masks produced by DSD-UNet, rather than with the segmentation-guided pipeline alone.

The stability of the segmented regions also ensures the algorithm delivers consistent and reliable results in challenging scenarios involving strong reflections, backlighting, or partial occlusion.

## 5 Conclusion

In this study, we propose a lightweight segmentation model, DSD-UNet, for orchard-based litchi harvesting tasks, aimed at improving both the accuracy and efficiency of clustered litchi fruit and stem segmentation. The model reconstructs the encoder using DS-Convs, substantially reducing model parameters and computational complexity. A dual cross-attention mechanism is integrated into the skip connections to enhance the extraction and fusion of fine stem-structure features. In addition, a hybrid loss function (BL-Loss), combining binary cross-entropy and LS-Loss, is employed at the prediction layer to alleviate within-foreground pixel imbalance and improve the boundary continuity of slender fruit–stem connections. Experimental results show that DSD-UNet achieves an *mIoU* of 91.40%, *mPA* of 98.95%, *Precision* of 95.73%, *Recall* of 95.37%, and *F1-score* of 95.42%. The inference speed reaches 15.41 *FPS*, with only 5.16M parameters, fully meeting the requirements for both accuracy and real-time application. Compared with mainstream methods including U-Net, CMTFNet, HRNet, PSPNet, Segformer, Fast-SCNN, PIDNet-Lite, MaSSFormer, and YOLO11n-seg, DSD-UNet outperforms them by 3.08, 3.60, 8.62, 5.06, 11.13, 6.46, 9.47, 5.86, and 13.12 percentage points in *mIoU*, respectively. Moreover, DSD-UNet achieves the best performance across key metrics including *mIoU*, *Precision*, *F1-score*, *mPA*, and inference speed, with *Recall* and model size also maintaining competitive performance, thereby validating the comprehensive effectiveness of the proposed improvements.

This study provides a solid foundation for intelligent perception and segmentation technologies in robotic litchi harvesting, and the proposed segmentation framework may also serve as a reference for automated harvesting of other fruits and vegetables with similar structural and growth characteristics. Despite the excellent performance of the proposed approach in segmenting litchi fruits and stems, issues such as segmentation errors and reduced accuracy persist under extreme illumination conditions (e.g., very low-light scenarios). Future research will focus on developing illumination-invariant feature extraction methods and integrating multi-modal information (such as 3D point clouds) to further enhance the robustness and generalization capability of the model in diverse real-world environments.

## Data Availability

The raw data supporting the conclusions of this article will be made available by the author, without undue reservation.
